# Robotic Rehabilitation: An Opportunity to Improve Cognitive Functions in Subjects With Stroke. An Explorative Study

**DOI:** 10.3389/fneur.2020.588285

**Published:** 2020-11-19

**Authors:** Irene Aprile, Giulia Guardati, Valeria Cipollini, Dionysia Papadopoulou, Alessia Mastrorosa, Letizia Castelli, Serena Monteleone, Alessandra Redolfi, Silvia Galeri, Marco Germanotta

**Affiliations:** ^1^IRCCS Fondazione Don Carlo Gnocchi ONLUS, Florence, Italy; ^2^Fondazione Policlinico Universitario A. Gemelli IRCCS, UOC Neuroriabilitazione ad Alta Intensità, Rome, Italy; ^3^IRCCS Fondazione Don Carlo Gnocchi ONLUS, Milan, Italy

**Keywords:** rehabilitation, robotics, stroke, executive function, attention, memory

## Abstract

**Background:** After a stroke, up to three-quarters of acute and subacute stroke survivors exhibit cognitive impairment, with a significant impact on functional recovery, quality of life, and social engagement. Robotic therapy has shown its effectiveness on motor recovery, but its effectiveness on cognitive recovery has not fully investigated.

**Objective:** This study aims to assess the impact of a technological rehabilitation intervention on cognitive functions in patients with stroke, using a set of three robots and one sensor-based device for upper limb rehabilitation.

**Methods:** This is a pilot study in which 51 patients were enrolled. An upper limb rehabilitation program was performed using three robots and one sensor-based device. The intervention comprised motor/cognitive exercises, especially selected among the available ones to train also cognitive functions. Patients underwent 30 rehabilitation sessions, each session lasting 45 minutes, 5 days a week. Patients were assessed before and after the treatment with several cognitive tests (Oxford Cognitive Scale, Symbol Digit Modalities Test, Digit Span, Rey–Osterrieth Complex Figure, Tower of London, and Stroop test). In addition, motor (Fugl–Meyer Assessment and Motricity Index) and disability (modified Barthel Index) scales were used.

**Results:** According to the Oxford Cognitive Scale domains, a significant percentage of patients exhibited cognitive deficits. Excluding *perception* (with only one patient impaired), the domain with the lowest percentage of patients showing a pathological score was *praxis* (about 25%), while the highest percentage of impaired patients was found in *calculation* (about 70%). After the treatment, patients improved in all the investigated cognitive domains, as measured by the selected cognitive assessment scales. Moreover, motor and disability scales confirmed the efficacy of robotics on upper limb rehabilitation in patients with stroke.

**Conclusions:** This explorative study suggests that robotic technology can be used to combine motor and cognitive exercises in a unique treatment session.

**Clinical Trial Registration:** www.ClinicalTrials.gov, identifier: NCT04164381.

## Introduction

Cognitive dysfunctions are common consequences of stroke (1, 2). The reported percentages of patients with cognitive impairment after stroke are variable (3) and depend on several aspects, such as the inclusion of recurrent strokes, time of evaluation after stroke, dementia criteria, and exclusion of aphasic patients (4). It is estimated that up to three-quarters of acute and subacute stroke survivors exhibit cognitive impairment (5, 6). Cognitive impairment can significantly compromise functional recovery, quality of life, and social engagement after stroke (6–8). Indeed, some authors showed that the impairment of the cognitive functions can negatively influence rehabilitation strategies (9) and be a negative predictor of functional and motor outcomes after upper limb robotic therapy in patients with stroke (10).

Robotic therapy has been proposed as a viable approach for the rehabilitation of the upper limb, as a way to increase the amount and the intensity of the therapy, and to standardize the treatment (11). The most recent meta-analysis suggests that robotics can improve upper limb motor function and muscle strength after stroke (12), and, when compared to a similar amount of conventional therapy, no significant differences in terms of motor recovery are detected (13, 14).

On the contrary, to the best of our knowledge, the efficacy of robotics in restoring cognitive deficits was never explored. Robotic and technological devices can present a variety of solutions with different levels of technology, in terms of mechanical structure, level of assistance, and complexity of exercises. Even though the first devices were pretty basic in terms of rehabilitation scenario and required tasks, nowadays the implementation of new graphical interfaces and more ecological scenarios, as well as more cognitively demanding tasks, can allow an active physical and cognitive engagement of patients during robotic therapy. This can be promoted through adaptive assistance (15), to promote patient's engagement (16), as well as through cognitive challenge (17), automated task difficulty adaptation (18, 19), and motivating visual and auditory feedback (20). Feedback about movement performance not only enhances motivation but also facilitates plasticity in the motor cortex if it arrives synchronously with the motor output (21), promoting the mechanisms of connectivity remodulation (22).

Therefore, we hypothesized that a robotic treatment, based on the execution of exercises, specifically selected, based on concurrent motor/cognitive tasks can improve cognitive deficits beyond motor function in patients with stroke. The current study is an explorative study aimed to evaluate the effects of upper limb robotic rehabilitation training on the cognitive functions of subacute stroke patients.

## Materials and Methods

### Study Design and Participants

In this pilot study we recruited a sample of consecutive subjects with (a) a single ischemic or hemorrhagic stroke (verified by MRI or CT), (b) age between 35 and 85 years, (c) a time since stroke within 6 months, (d) cognitive abilities adequate to understand the experiments and follow instructions (Token test corrected by age and school level ≥26.5), and (e) upper limb impairment (Fugl–Meyer Assessment score ≤58). We excluded patients with (a) a history of recurrent stroke, (b) behavioral and cognitive disorders and/or reduced compliance, (c) fixed contraction in the affected limb (ankylosis, Modified Ashworth Scale equal to 4), and (d) severe deficits in visual acuity. The study was conducted following the International Conference on Harmonization Good Clinical practice guidelines and the Declaration of Helsinki. All participants gave written informed consent before study participation. The institutional Ethics and Experimental Research Committee approved the study protocol on March 13, 2019 (FDG_13.3.2019) that was registered on Clinicaltrial.gov (ClinicalTrials.gov Identifier: NCT04164381).

### Assessment

Demographic, anamnestic, and clinical data were recorded before the treatment (T0). Cognitive functions, upper limb performance, and dependence in activities of daily living were assessed at T0 and after the robotic rehabilitation intervention (T1).

### Cognitive Assessment

As a cognitive screening tool, we used the Italian version of the *Oxford Cognitive Screen* (OCS), recently developed with the specific aim to describe the cognitive deficits after stroke (23, 24). The scale consists of 10 tasks encompassing five cognitive domains: attention and executive function, language, memory, number processing, and praxis. Furthermore, it includes a brief evaluation of visual field defects.

The effects of robotic rehabilitation on cognitive functions were explored using specific tools, in addition to the OCS. The cognitive assessment lasted about 90 minutes; sometimes, two sessions were requested to conclude the tests. Specifically, the tests listed below were used.

#### Symbol Digit Modalities Test (Attention and Processing Speed)

It is an easily administered test for overall neurocognitive and executive functioning including attention, planning, and organizing in addition to visual scanning, and motor speed. The subject is presented with a page where, in the first row, nine symbols are one-to-one associated with nine digits, from 1 to 9. Then, the rows below contain only symbols, and subjects are required to orally report the digit associates with each symbol. The number of correct responses in 90 seconds is measured. A higher score indicates higher cognitive functions (25, 26).

#### Digit Span Task (Memory)

We used the Digit span forward task originally proposed by Hebb (27). The examiner pronounces a list of digits, at a rate of approximately one digit per second, and the subjects are required to immediately repeat the list in the same order. If they succeed, a list one digit longer is presented. If they fail, a second list of the same length is presented. If subjects are successful on the second list, a list one digit longer is given, as before. However, if subjects also fail on the second list, the test is ended. The length of the digit sequences gradually increases, starting with a sequence of three numbers (e.g., 5, 8, 2) to a sequence of a maximum of nine items (e.g., 7, 1, 3, 9, 4, 2, 5, 6, 8). The span is established as the length of the longest list correctly recalled (28).

#### Rey–Osterrieth Complex Figure (Visuospatial Abilities and Visual Memory)

The task, originally designed by Rey (29) and later standardized by Osterrieth (30), requires the subject to copy a complex geometrical figure (immediate copy condition) (29, 31). For the test, performance accuracy was calculated by applying the standard scoring criteria, in which the geometrical figure is divided into 18 units and scored on a 2-point scale for both accuracy and placement (32).

#### Tower of London (Executive Functions)

It is a useful neuropsychological instrument to measure planning and problem-solving abilities (33–36). Briefly, it consists of a board with three vertical pegs of different increasing length in which three different wooden balls of different colors are placed. The shortest peg only accommodates one ball, the second two, and the third three. Subjects are presented with a given configuration of balls inside the pegs and a picture of the final configuration. Subjects are then required to move the balls to reach the final configuration, without breaking some rules (each peg can accommodate a different number of balls, just one ball might be moved at a time, the balls cannot be placed outside the pegs, and a maximum number of moves is allowed). In this study, three scores were computed: *points, time* (measured as the sum of the planning and the execution time), and *errors* (36).

#### Stroop Color and Word Test (Executive Functions)

It is a neuropsychological tool widely used in clinical practice to assess selective attention, cognitive flexibility, and sensitivity to interference, abilities that have been linked to the frontal lobes. We used the short version (37) in which three tasks are proposed: (1) *word* (word reading)−3 lists of 10 words (“red,” “blue,” “green”) are provided in random order to the patients, each written with black ink; they must read the written words; (2) *color* (color designation)−3 lists of 10 colored (red, blue, green) circles are provided in random order to the patients; they must name the color of the circles; and (3) *color–word* (interference test)−3 lists of 10 words (“red,” “blue,” “green”), each written with colored ink (red, blue, or green) different from the name of the color indicated by the word, in all possible combinations, are proposed to the patients in random order, and they are asked to name the color of the ink used to write the word, not the word itself. For each test, the execution time (T1, T2, and T3) and any errors made are recorded. Two interference effects are then calculated and used as outcomes: *time* (difference between the time spent in the third test and the average time spent in the two previous tasks) and *error* (difference between the number of errors made in the third test and the average time spent in the two previous tasks).

### Upper Limb Motor Performance and Activities of Daily Living Dependence

The effects of the rehabilitation were evaluated using also the following outcome measures: the Fugl–Meyer Assessment for Upper Extremity (FMA-UE) (38), to evaluate motor function; the upper-extremity subscale of the Motricity Index (39), to evaluate upper limb muscle strength; and the Modified Barthel Index (40), to evaluate activities of daily living and mobility.

### Treatment

**Figure 1:**
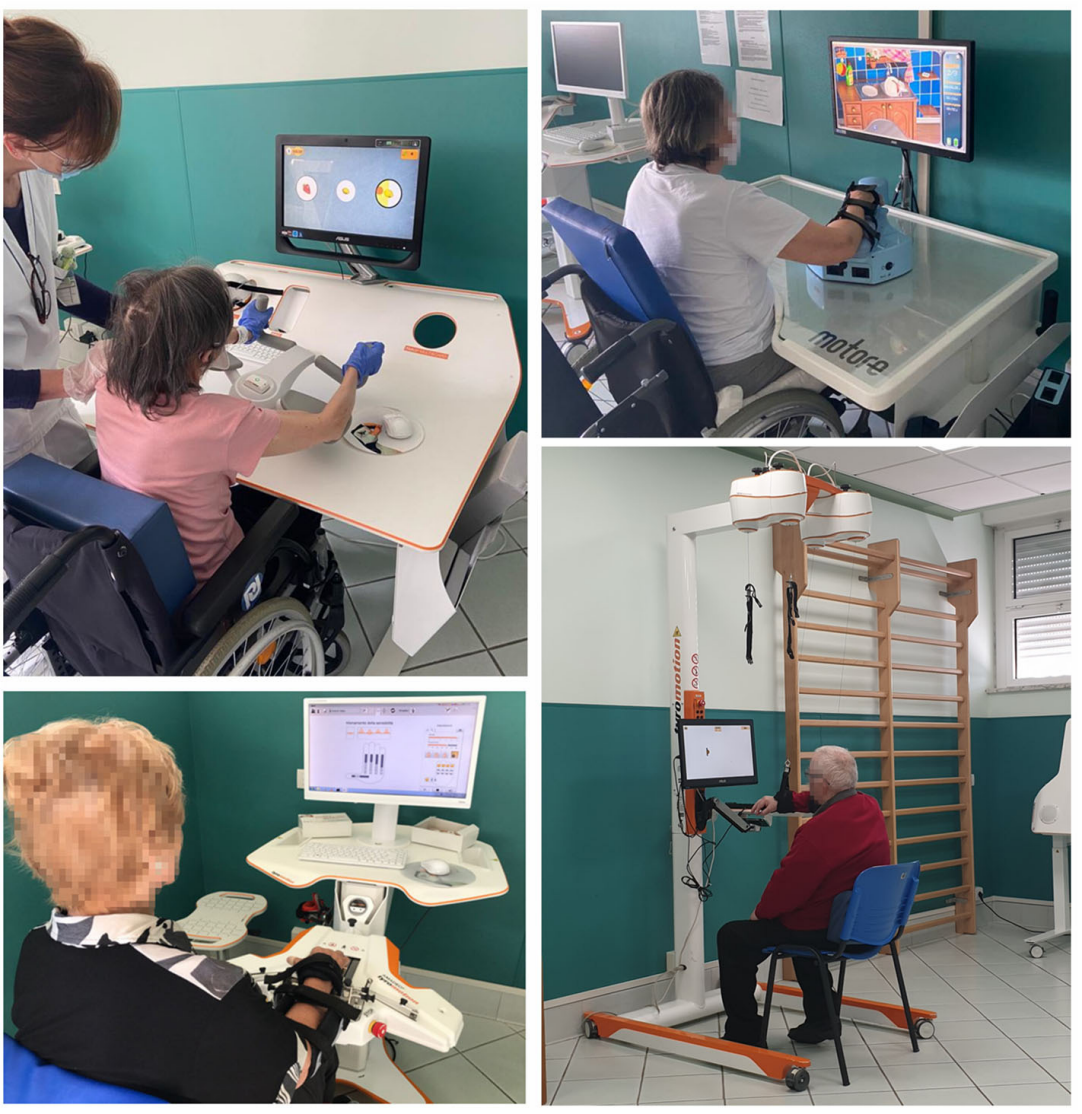
The robotic set: Pablo (upper left), Amadeo (lower left), and Diego (lower right) from Tyromotion and Motore (upper right) from Humanware.

Patients were treated with a set of three robots (i.e., with motors: Motore, Amadeo, and Diego) and one sensor-based device (i.e., without motors: Pablo) shown in Figure 1 (41, 42). The treatment was performed daily for 45 minutes, 5 days a week, for 30 sessions.

Motore (Humanware) is a robotic device that allows passive, active, and active-assistive planar movements of the shoulder and elbow joints. Amadeo (Tyromotion) is a robotic device that allows passive, active, and active-assistive finger flexion and extension movements. Pablo (Tyromotion) is a device based on a handle equipped with two sensors (a dynamometer and an inertial measurement unit), able to record the movement of the hand in the space and the forces applied to it but not to provide motorized assistance. The tasks require to perform unimanual or bimanual three-dimensional movements of the shoulder, elbow, and wrist or to apply forces to the handle; bimanual movement are performed through two additional tools, namely, the multiboard and the multiball (14). Diego (Tyromotion) is a robotic system that allows three-dimensional, unimanual, and bimanual movements of the shoulder joint, with arm weight support.

**Figure 2:**
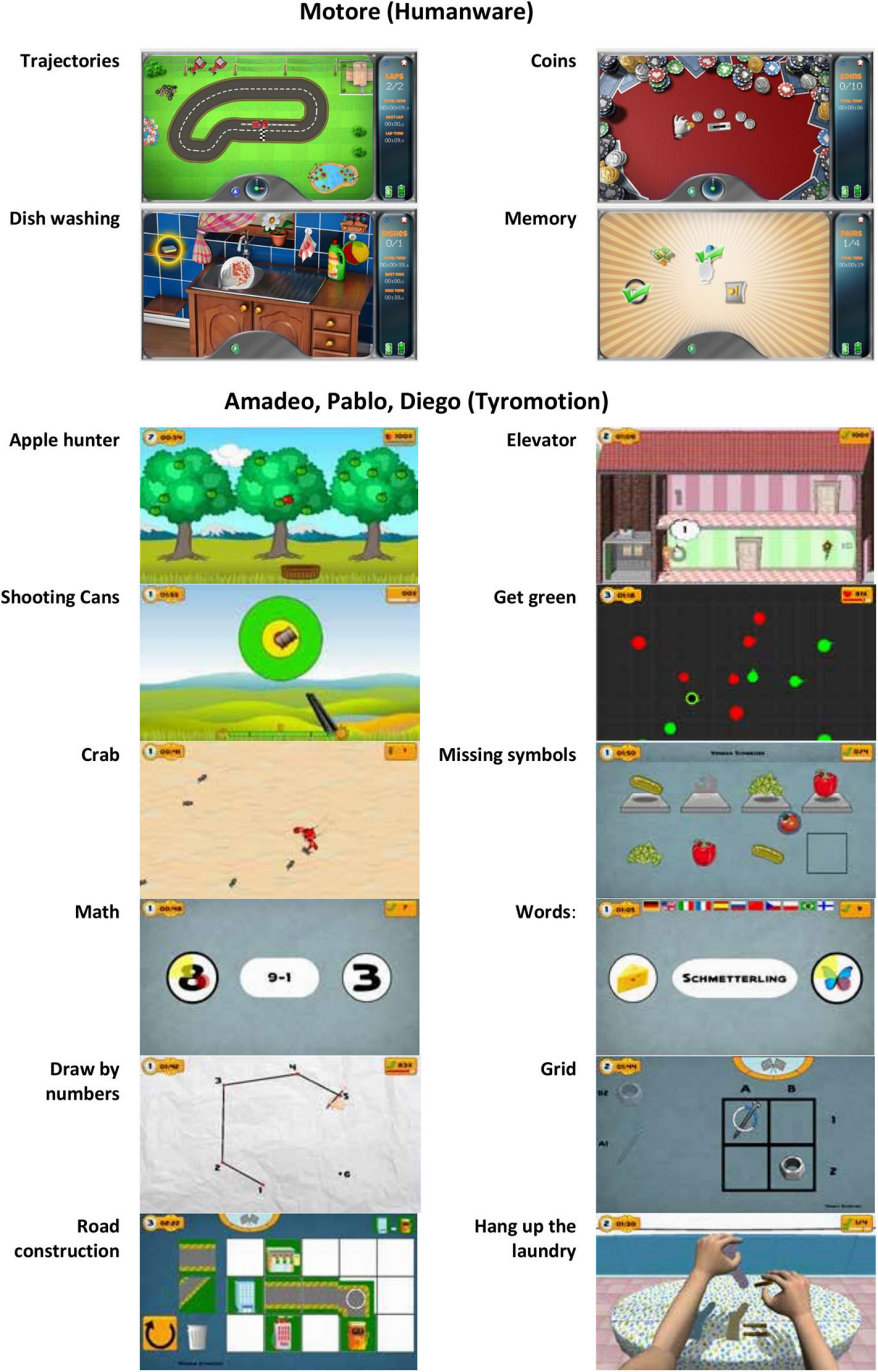
List of motor/cognitive exercises performed with the devices.

During the treatment, patients performed both motor and cognitive tasks, and the devices provided visual and auditory feedback to help them. In particular, a set of motor/cognitive exercises was selected among those available in the robotic devices to train attention, memory, executive function, speed of processing, and visuospatial abilities (Figure 2). The rehabilitation program was focused on interactive games, performed through the support of the assistive forces provided by the robotic devices. In patients with mild impairment, it was also possible to reduce or remove this support, including in the intervention also motor/cognitive tasks performed without external help through the sensor-based device.

Specifically, using the robot Motore, the following exercises were executed:

- *Trajectories—*the patient is asked to drive his car along a track (training for visual scanning, attention, visuospatial ability);
- *Coins—*the patient is asked to identify and collect some golden coins arranged along an arc (while the others remain silver) and bring them back to the center of the worktop (training for visual scanning and attention);
- *Dishwashing—*this exercise simulates a daily life activity: the patient is asked to wash the dishes according to a pre-established sequence of actions, such as bring the plate into the sink, open the tap, reach the sponge, etc. (training for procedural memory, semantic memory, planning abilities, and attention);
- *Memory—*groups of icons are presented to the patient who is asked to identify and associate the icons (one by one) by meaning (training for memory).

Using Pablo, Diego, and Amadeo, the following exercises were executed (these devices shared the same software):

- *Applehunter—*falling apples (changing color from green—on the tree—to yellow—immediately before falling—to red) must be caught with a basket moved by the patient (training for coordination, selective attentiveness, processing speed, visual scanning);
- *Elevator*—the patient is asked to move an elevator in a building, with the aim of picking up people and taking them to the correct floor (training for concentration and attention, visuospatial ability, coordination, understanding numbers);
- *Shooting cans—*the patient is asked to pull a trigger to shoot the cans moving past a fixed reticule on the screen (training for concentration and attention, processing speed, visuospatial ability);
- *Get green*—the patient controls a dot and must guide it into the green circles while avoiding the red circles (training for responsiveness and processing speed, selective attentiveness);
- *Crab*—the patient controls the direction and the speed of a crab, running around on a beach; the goal is to catch as many of the ants, which try to run away from the crab (training for visuospatial ability and spatial orientation, processing speed, attention);
- *Missing symbols*—the patient has to move the device to select and place in the correct location the missing symbol (training for selective attentiveness and planning);
- *Math Mental*—solving of simple arithmetic problems and selecting the correct solution (training for calculus ability);
- *Words*—reading of simple words and assigning them to the respective symbols (training for reading and understanding ability).
- *Draw by numbers*—the patient controls the pen and must connect the dots in the correct order (training for visuospatial ability, number count ability, attention, visual scanning);
- *Grid*—place the symbols in the designated grid positions (training for attention, visuospatial ability, memory, visual scanning);
- *Road construction*—build a street between the buildings displayed on the upper right of the screen (training for visuo-spatial and constructive ability, planning);
- *Hang up the laundry*—laundry items and clothespins must be taken from the table and attached to the clothesline (training for planning).

For each device, the exercises were selected to target, during the 30-session rehabilitation intervention, all the investigated cognitive functions. Moreover, being differently demanding from a motor point of view, the exercises were also selected for each patient according to her/his severity, based on the FMA-UE score (43), as reported in Table 1. In addition, the level of difficulty for each exercise varied according to the patient's ability and improvement.

**Table 1:** List of motor/cognitive exercises performed with the set of devices, grouped according to the trained cognitive domain, and the availability for patients with different degree of severity, according to the Fugl–Meyer Assessment for Upper Extremity (FMA-UE): severe (FMA-UE 0–28), moderate (FMA-UE 29–42), and mild (FMA-UE 43–66) (43).

	**Severe**	**Moderate**	**Mild**
Attention Processing speed	Trajectories Coins Applehunter Elevator	Trajectories Coins Applehunter Elevator Get green Crab Missing symbols Draw by numbers Grid	Trajectories Coins Applehunter Elevator Get green Crab Missing symbols Draw by numbers Grid Shooting cans Math
Visuospatial ability	Trajectories Coins Applehunter Elevator	Trajectories Coins Applehunter Elevator Get green Crab Draw by numbers	Trajectories Coins Applehunter Elevator Get green Crab Draw by numbers Shooting cans Road construction
Memory	Washing dishes Memory	Washing dishes Memory Words Grid	Washing dishes Memory Words Grid
Executive Functions Planning	Washing dishes Elevator	Washing dishes Elevator Missing symbols	Washing dishes Missing symbols Math Road construction Hang up the laundry

During the treatment, a group of three subjects was supervised by one physiotherapist. During each session, the physiotherapist used one device for each patient to minimize the time required to move the subject from one system to another, but throughout the 30-session rehabilitation intervention, all the devices were used; with respect to the sensor-based device, patients with moderate or severe impairment performed bimanual task only, i.e., with the support of the unimpaired arm.

Further, patients underwent a comprehensive rehabilitation program including individual conventional physiotherapy (six times/week), lasting 45 minutes, focused on lower limbs, sitting and standing training, balance, and walking. Patients with language disorders performed speech training.

### Statistical Analysis

Visual inspection and Shapiro–Wilk test showed that data did not meet the criteria for parametric analysis, and therefore, non-parametric tests were used. Specifically, to assess the effects of the rehabilitation intervention on motor and cognitive domains, data obtained at T0 and T1 were compared by means of Wilcoxon signed-rank tests for numeric and ordinal data and the McNemar test for proportions. For all the statistical analyses, a p value of 0.05 was deemed significant. Statistical analysis was performed with SPSS (IBM SPSS Statistics for Windows, Version 25.0, Armonk, NY).

## Results

**Figure 3:**
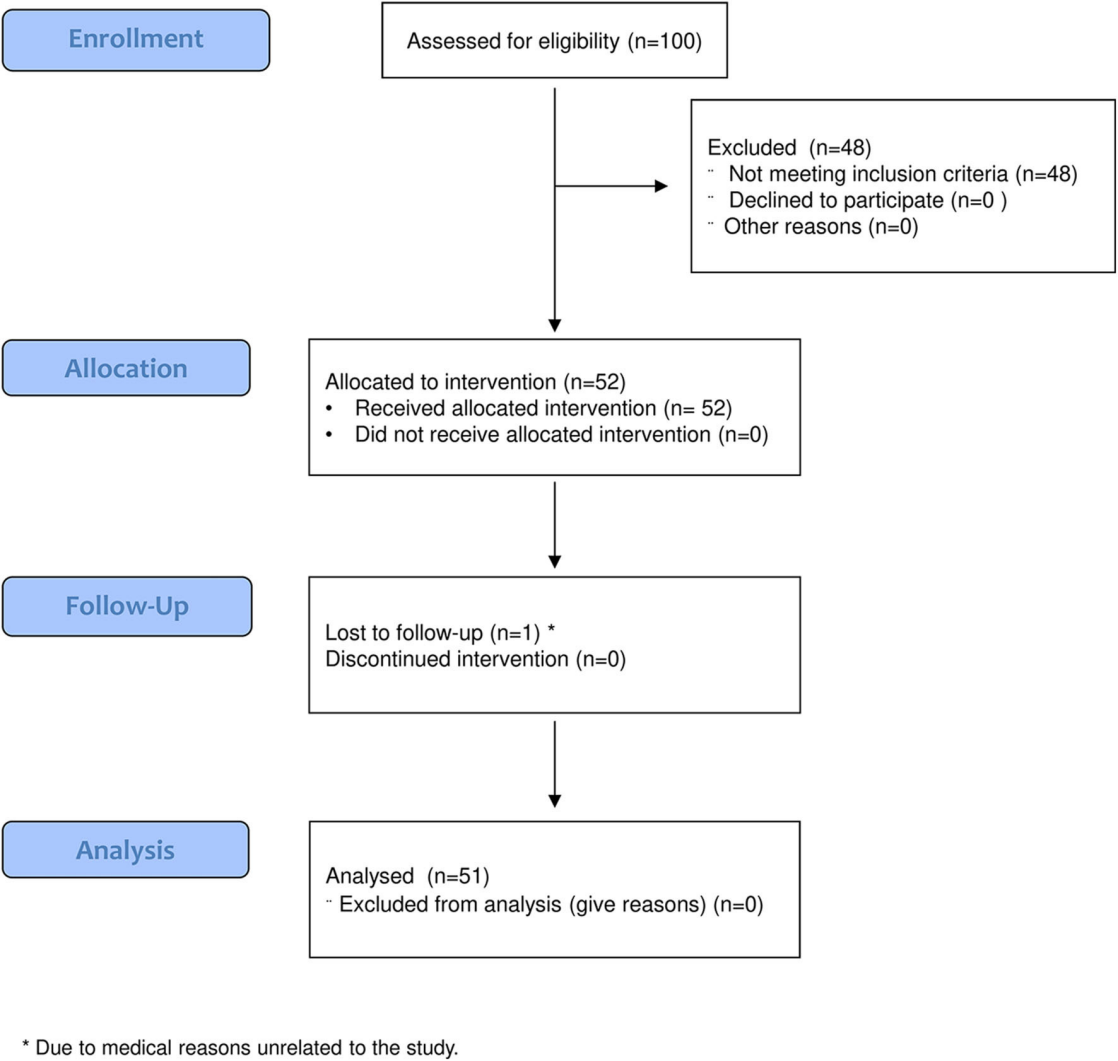
Flow chart of the study.

One hundred patients were assessed for eligibility, 48 of whom were excluded because of the inclusion criteria. Fifty-two patients were evaluated at T0 and received the allocated intervention. Of those, one patient did not undergo the follow-up evaluation, and therefore, 51 patients were evaluated at T1 and considered for the analysis (Figure 3). The demographic and clinical features of the analyzed sample are given in Table 2.

**Table 2:** Demographic and clinical characteristics of the analyzed sample (*N* = 51).

**Entry Characteristics**	
Age (years), mean (SD)	68.4 (12.4)
Sex, *n* (%)	
Men	29 (56.9%)
Women	22 (43.1%)
Education years, *n* (%)	
5	11 (21.6%)
8	15 (29.4%)
13	22 (43.1%)
18	3 (5.9%)
Index stroke type, *n* (%)	
Ischemic	36 (70.6%)
Hemorrhagic	15 (29.4%)
Dominant side, *n* (%)	
Right	47 (92.2%)
Left	4 (7.8%)
Affected side, *n* (%)	
Right	23 (45.1%)
Left	28 (54.9%)
Language impairment, *n* (%)	11 (21.6%)
Neglect syndrome, *n* (%)	10 (19.6%)
Days from index stroke to enrollment, mean (SD)	74.6 (41.3)

**Figure 4:**
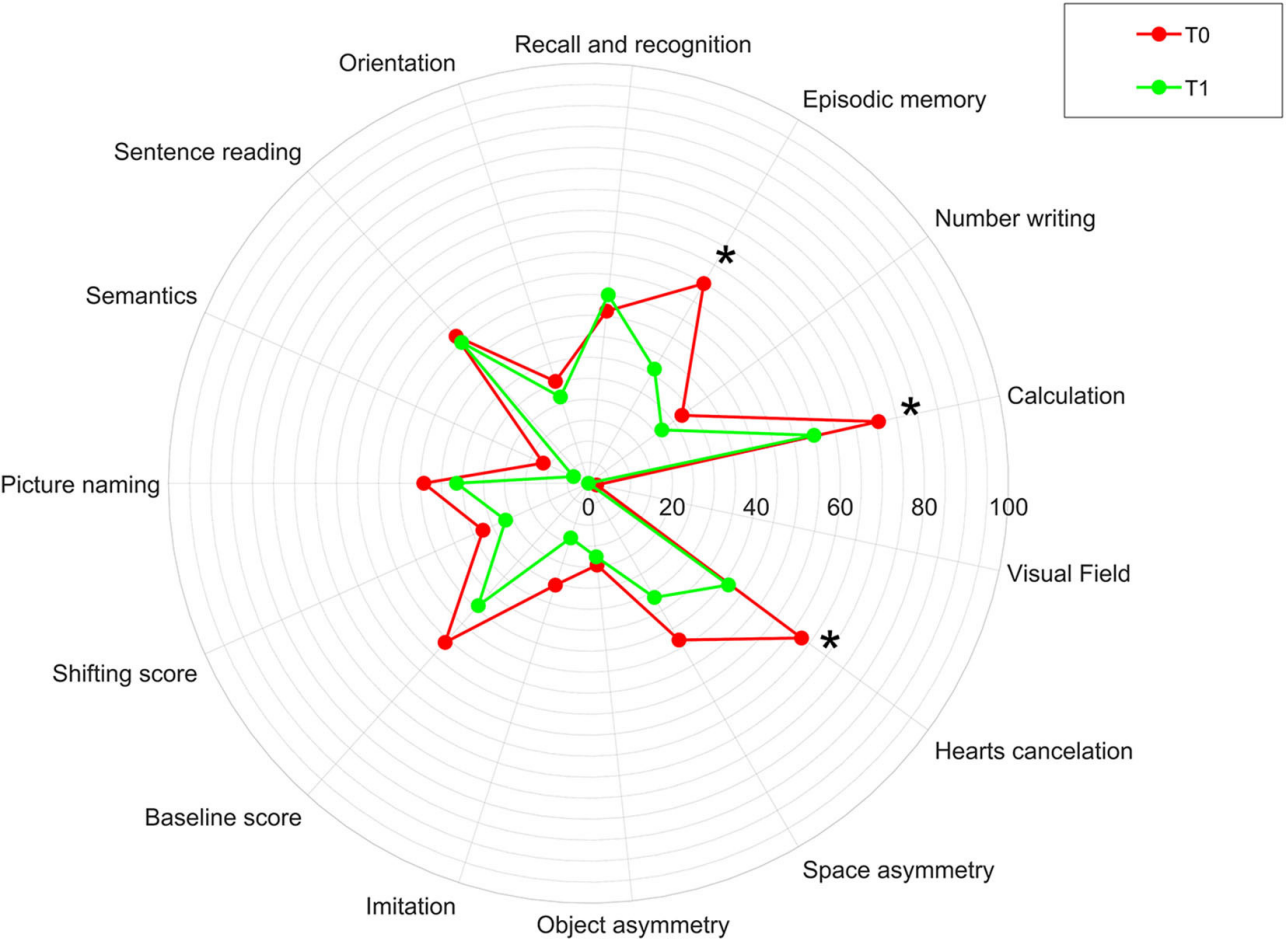
Percentage of patients obtaining a pathological score in the Oxford Cognitive Screen (OCS) in our sample before (T0) and after (T1) the rehabilitation treatment. The asterisk indicates a statistically significant difference between T0 and T1 in our sample.

Figure 4 shows the percentages of patients obtaining a pathological score in the Oxford Cognitive Screen before and after the treatment. In particular, pathological scores have been found in *language and memory* domains in about half of the cases, *number domains* and in particular calculation function in 70.6% of the cases and number writing in the 27.5% of the cases, *perception* (visual field) in only one case, *spatial attention* in about 60% of the cases, *praxis* in about 25% of the cases, and e*xecutive function* in about 50% of the cases. After treatment, the percentage of patients obtaining a pathological score in the OCS subscore significantly reduced in the episodic memory (*p* = 0.008), calculation (*p* = 0.021), and visual attention (heart cancelation task, *p* = 0.001) fields.

**Figure 5:**
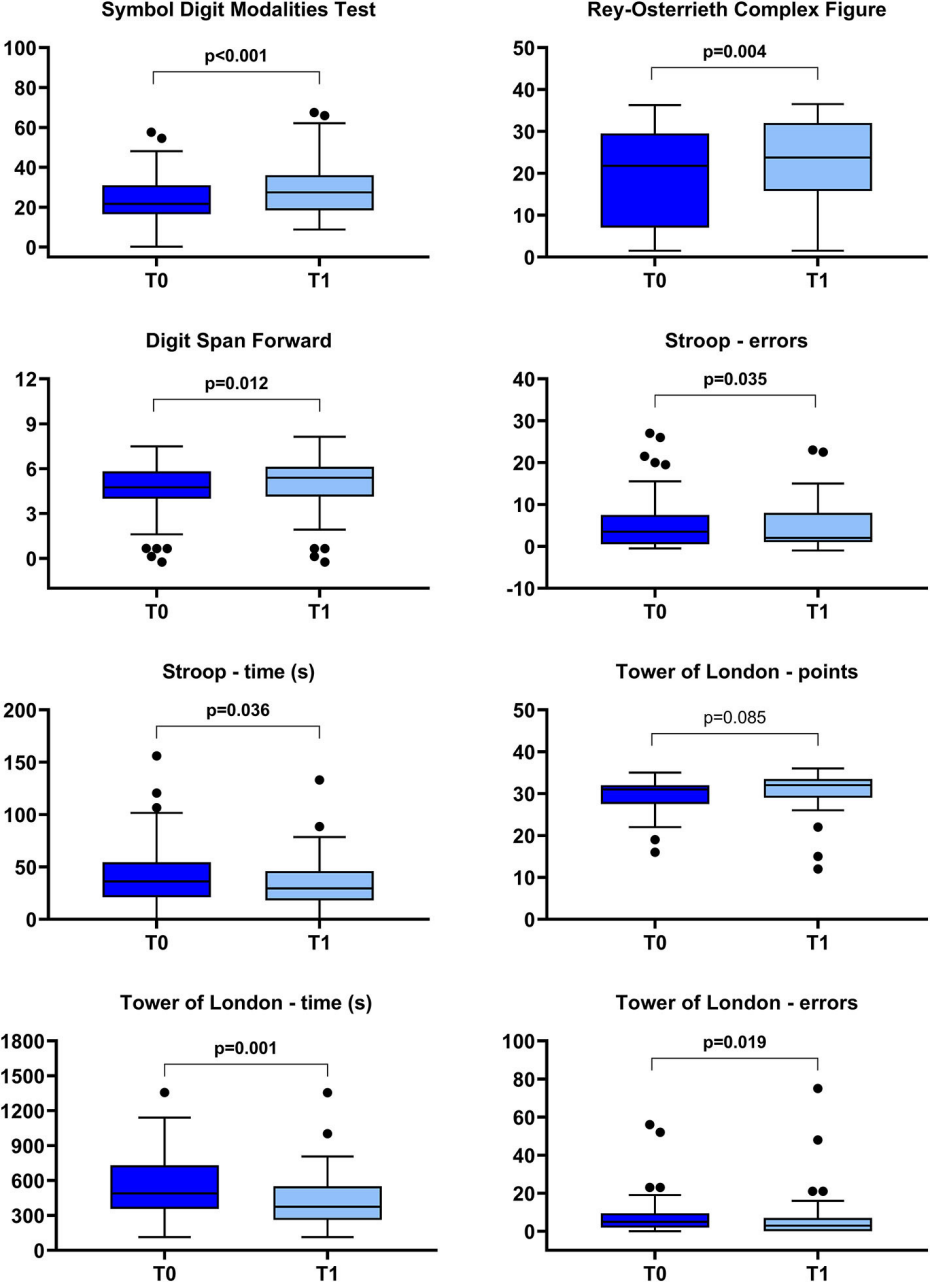
Box-plot diagrams showing the scores obtained before (T0) and after (T1) the robotic treatment in the cognitive tests assessing attention and processing speed (*Symbol Digit Modalities Test*), visuospatial abilities and visual memory (*Rey–Osterrieth Complex Figure*), memory (*Digit Span*), and executive functions (Stroop and Tower of London tests). The boxes show the interquartile range (IQR, from the 25th to the 75th percentile). The horizontal line within each box indicates the median. The vertical bars (whiskers) indicate the range of observations excluding outliers. Dots represent outliers, i.e., observations higher than the 75th percentile plus 1.5 times IQR or lower than the 25th percentile minus 1.5 times IQR. *P* values refer to the Wilcoxon signed-rank test and are marked in bold when a statistically significant difference at *p* < 0.05 level between T0 and T1 was detected.

In Figure 5, the changes in cognitive functions, as measured by the selected outcome measures, are reported. A statistically significant improvement was found in all the investigated domain: attention and processing speed (Symbol Digit Modalities Test), memory (Digit Span score), visuospatial abilities and visual memory (Rey–Osterrieth complex figure), and executive functions (Stroop errors and time, Tower of London error and time). Only the subscore “points” of the Tower of London Test did not significantly change.

Regarding the dependence on activities of daily living, the sample showed a T0 a severe disability, as measured using the modified Barthel Index, associated with a moderate to severe impairment in upper limb motor functions and strength (as measured by the Fugl–Meyer Assessment and the Motricity Index, respectively). Table 3 shows the effects of the robotic treatment on the upper limb motor performance and daily living activities. In particular, after the treatment, a significant improvement was observed in upper limb impairment, measured using the Fugl–Meyer Assessment (mean change, 11.9 ± 10.1; *p* < 0.001); upper limb muscle strength, as measured by the Motricity Index (mean change, 16.2 ± 12.9; *p* < 0.001); and ability in activities daily living, as shown by the modified Barthel Index (mean change, 22.6 ± 15.5; *p* < 0.001).

**Table 3:** Motor and cognitive assessment scores before (T0) and after the robotic treatment (T1), together with the p values of the Wilcoxon signed-rank test (with values in bold indicating a statistically significant difference between T0 and T1).

**Investigated domain**	**Measure**	**T0**		**T1**		**Change from baseline**		***p***
		**Mean**	**SD**	**Mean**	**SD**	**Mean**	**SD**	
Ability	Barthel Index (*N* = 51)	40.3	18.3	62.8	24.0	22.6	15.5	**<0.001**
Muscle strength	Motricity Index (*N* = 51)	37.3	27.9	53.5	29.0	16.2	12.9	**<0.001**
Impairment	Fugl–Meyer (*N* = 51)	21.5	18.1	33.4	21.0	11.9	10.1	**<0.001**

## Discussion

The improvement in cognitive functions is among the top 10 research priorities relating to life after stroke, according to a consensus from stroke survivors, caregivers, and health professionals (2). Moreover, cognitive impairment was considered as a priority in the rehabilitation path of patients after stroke (44) because it influences the recovery of the motor function and ability in life daily activities. In the last Cochrane Review on cognitive rehabilitation for attention deficits following stroke (45), the authors highlight that improving attention, also in the short term, is very important during motor and functional rehabilitation program because high attention may enable people to engage better the exercises proposed with a high ability to cope with proposed tasks.

Several digital applications have been developed to train cognitive deficits. Some authors reported that an interactive virtual training is a useful treatment capable of stimulating cognitive abilities (amnesic-attentive functions and visuospatial cognition), executive processes, and behavioral abilities in patients with neurological disorders (46). In general, the advantage of incorporating virtual reality into rehabilitative programs is to create a positive learning experience that can also be fun and motivating for the patient (47). These virtual reality programs, developed to increase the patient's engagement, can contain cognitive exercises, and, sometimes, they can be integrated into robotic devices designed for motor rehabilitation. Therefore, rehabilitation robotics in the last years has included virtual reality programs and exercises stimulating cognitive functions, which can be proposed and performed during motor exercises. Nevertheless, usually, the aim of the robotic treatment is the improvement in motor performance and activities of daily living, while the cognitive deficits are often ignored or treated independently from motor impairment (48).

A cognitive treatment is crucial for the subjects in which cognitive and motor impairments are often present at the same time, as stroke patients (49). Indeed, the limited transfer of upper limb motor improvement in upper limb motor ability to different domains, as the activities of daily living, observed in several studies (50), could be due to the lack of attention toward the coexistent cognitive impairment. Few studies explored the cognitive effects of a robotic rehabilitation program (51, 52), and they did not use tools to investigate specific cognitive functions.

This is the first pilot study in which cognitive training and upper limb motor rehabilitation are combined thanks to the use of robotic and sensor-based devices on a sample of subacute stroke patients, using a cognitive screening tool and a set of cognitive outcome measures investigating attention and processing speed, memory, visuospatial abilities, visual memory, and executive functions. As a cognitive screening tool, we choose to use the Oxford Cognitive Screening because our group was part of the Italian OCS Group and participated in the study detecting cognitive impairment in Stroke patients using OCS, so an adequate training to the administration of OCS was performed to our researchers. The OCS, even if this is a simple cognitive screening tool, showed that, after robotic treatment, our patients significantly improved in spatial attention, episodic memory, and calculation.

Interesting results emerged when a battery of specific cognitive tools was used to test specific cognitive domains. After upper limb robotic treatment, all the explored cognitive domains significantly improved, in particular attention and processing speed, visuospatial abilities visual memory, executive functions, and memory. Then, this explorative study shows preliminary but encouraging data on the opportunity offered by robotic technology to combine motor and cognitive exercises in a unique treatment session. Note that we have selected the cognitive domain to be investigated and, therefore, the cognitive measures based on the exercises available in our set of robots and sensor-based devices. Physicians and physiotherapists need to identify specific cognitive exercises that are feasible using the robots and the technologies that are available in their rehabilitation ward. In this sense, it is also important to adopt the correct cognitive assessment tools able to intercept the possible change in the targeted cognitive fields. In our work, the use of a cognitive screening tool, together with a pool of specific cognitive tools, could seem redundant, but our aims were (a) to characterized our sample in term of general cognitive decline and then (b) to evaluate the improvement in some specific cognitive functions, which are the target of our robotic rehabilitation.

The proposed approach can be a resource to a more efficient rehabilitation treatment because it permits to treat at the same time two aspects often impaired in stroke patients; however, it is important to consider that this approach is feasible only if some requirements are satisfied: (a) the devices must include motor exercises specifically designed to stimulate cognitive functions (as visual memory, processing speed, etc.) and (b) the presence of a multidisciplinary team, made of neuropsychologists, physiatrists, physiotherapists, and speech therapists, with expertise in robotic rehabilitation, working synergistically on a new vision for the robotic rehabilitation.

The main limitation of this study is the lack of a control group, and therefore, the results of this pilot study have to be considered as a starting point that certainly encourages us to better use the potentiality of robotics and technologies. In the light of the above-mentioned limit, it is not possible to exclude that the cognitive functions here explored have improved spontaneously or because of the conventional rehabilitation that our patients performed in addition to the upper limb robotic rehabilitation. However, in a previous study in which the responsiveness and predictive validity of the Tablet-Based Symbol Digit Modalities Test was tested in a sample of 50 stroke patients undergoing a rehabilitation treatment (53), the authors found an increment of 3.3 points on the Symbol Digit Modalities Test, lower than the improvement that we observed in our sample (5.6 points). Indeed, this result suggests a beneficial effect of the proposed robotic intervention. Unfortunately, we did not find similar studies using the other cognitive tools proposed in our study (as the Rey–Osterrieth Complex Figure, the Digit Span, the Stroop, or the Tower of London tests) to compare our results with. Moreover, this study has investigated the combined effect of robotic and sensor-based devices on cognitive rehabilitation, so the specificity of the result in relation to each type of intervention (i.e., robotic vs. sensor-based vs. traditional treatment) is hard to establish. To better investigate the efficacy of the cognitive exercises administered using robotic or sensor-based devices (within motor rehabilitation program) compared to cognitive exercises administered using conventional methods, further studies, and randomized clinical trials, should be designed.

## Data Availability Statement

The raw data supporting the conclusions of this article are available from the corresponding author on reasonable request.

## Ethics Statement

The studies involving human participants were reviewed and approved by Comitato Etico della Sezione IRCCS Fondazione Don Carlo Gnocchi del Comitato Etico IRCCS Regione Lombardia. The patients/participants provided their written informed consent to participate in this study.

## Author Contributions

Conceptualization was carried out by IA, SG, and MG. IA, SG, and MG implemented the methodology. MG analyzed the data. GG, VC, DP, AM, LC, SM, and AR performed the investigation. IA and MG wrote and prepared the original draft. All authors reviewed and edited the manuscript.

## Conflict of Interest

The authors declare that the research was conducted in the absence of any commercial or financial relationships that could be construed as a potential conflict of interest.

## References

[1] Gottesman RF Hillis AE. Predictors and assessment of cognitive dysfunction resulting from ischaemic stroke. Lancet Neurol. (2010) 9:895–905. 10.1016/S1474-4422(10)70164-2 20723846 PMC3592203

[2] Pollock A St George B Fenton M Firkins L. Top 10 research priorities relating to life after stroke - consensus from stroke survivors, caregivers, and health professionals. Int J Stroke. (2014) 9:313–20. 10.1111/j.1747-4949.2012.00942.x 23227818

[3] Lamb F Anderson J Saling M Dewey H. Predictors of subjective cognitive complaint in postacute older adult stroke patients. Arch Phys Med Rehabil. (2013) 94:1747–52. 10.1016/j.apmr.2013.02.026 23529143

[4] Pendlebury ST Rothwell PM. Prevalence, incidence, and factors associated with pre-stroke and post-stroke dementia: a systematic review and meta-analysis. Lancet Neurol. (2009) 8:1006–18. 10.1016/S1474-4422(09)70236-4 19782001

[5] Leśniak M Bak T Czepiel W Seniów J Członkowska A. Frequency and prognostic value of cognitive disorders in stroke patients. Dement Geriatr Cogn Disord. (2008) 26:356–63. 10.1159/000162262 18852488

[6] Nys GMS Van Zandvoort MJE De Kort PLM Van Der Worp HB Jansen BPW Algra A. The prognostic value of domain-specific cognitive abilities in acute first-ever stroke. Neurology. (2005) 64:821–7. 10.1212/01.WNL.0000152984.28420.5A 15753416

[7] Hochstenbach JB Anderson PG van Limbeek J Mulder TT. Is there a relation between neuropsychologic variables and quality of life after stroke? Arch Phys Med Rehabil. (2001) 82:1360–6. 10.1053/apmr.2001.25970 11588738

[8] Hommel M Miguel ST Naegele B Gonnet N Jaillard A. Cognitive determinants of social functioning after a first ever mild to moderate stroke at vocational age. J Neurol Neurosurg Psychiatr. (2009) 80:876–880. 10.1136/jnnp.2008.169672 19357128

[9] Chen C Leys D Esquenazi A. The interaction between neuropsychological and motor deficits in patients after stroke. Neurology. (2013) 80:S27–34. 10.1212/WNL.0b013e3182762569 23319483

[10] Leem MJ Kim GS Kim KH Yi TI Moon HI. Predictors of functional and motor outcomes following upper limb robot-assisted therapy after stroke. Int J Rehabil Res. (2019) 42:223–8. 10.1097/MRR.0000000000000349 30932930

[11] Gassert R Dietz V. Rehabilitation robots for the treatment of sensorimotor deficits: A neurophysiological perspective. J Neuroeng Rehabil. (2018) 15:46. 10.1186/s12984-018-0383-x 29866106 PMC5987585

[12] Mehrholz J Pohl M Platz T Kugler J Elsner B. Electromechanical and robot-assisted arm training for improving activities of daily living, arm function, and arm muscle strength after stroke. Cochrane Database Syst Rev. (2018) 9:CD006876. 10.1002/14651858.CD006876.pub5 30175845 PMC6513114

[13] Rodgers H Bosomworth H Krebs HI van Wijck F Howel D Wilson N. Robot assisted training for the upper limb after stroke. (RATULS): a multicentre randomised controlled trial. Lancet. (2019) 394:51–62. 10.1016/S0140-6736(19)31055-4 31128926 PMC6620612

[14] Aprile I Germanotta M Cruciani A Loreti S Pecchioli C Cecchi F. Upper limb robotic rehabilitation after stroke: a multicenter, randomized clinical trial. J Neurol Phys Ther. (2020) 44:3–14. 10.1097/NPT.0000000000000295 31834217

[15] Riener R Lünenburger L Colombo G. Human-centered robotics applied to gait training and assessment. J Rehabil Res Dev. (2006) 43:679–94. 10.1682/JRRD.2005.02.0046 17123208

[16] Marchal-Crespo L McHughen S Cramer SC Reinkensmeyer DJ. The effect of haptic guidance, aging, and initial skill level on motor learning of a steering task. Exp Brain Res. (2010) 201:209–20. 10.1007/s00221-009-2026-8 19820920 PMC2832903

[17] Metzger JC Lambercy O Califfi A Conti FM Gassert R. Neurocognitive robot-assisted therapy of hand function. IEEE Trans Haptics. (2014) 7:140–9. 10.1109/TOH.2013.72 24968378

[18] Metzger JC Lambercy O Califfi A Dinacci D Petrillo C Rossi P Conti FM Gassert R. Assessment-driven selection and adaptation of exercise difficulty in robot-assisted therapy: a pilot study with a hand rehabilitation robot. J Neuroeng Rehabil. (2014) 11:154. 10.1186/1743-0003-11-154 25399249 PMC4273449

[19] Zimmerli L Krewer C Gassert R Müller F Riener R Lünenburger L. Validation of a mechanism to balance exercise difficulty in robot-assisted upper-extremity rehabilitation after stroke. J Neuroeng Rehabil. (2012) 9:6. 10.1186/1743-0003-9-6 22304989 PMC3286404

[20] Saposnik G Levin M. Virtual reality in stroke rehabilitation: A meta-analysis and implications for clinicians. Stroke. (2011) 42:1380–6. 10.1161/STROKEAHA.110.605451 21474804

[21] Stefan K. Induction of plasticity in the human motor cortex by paired associative stimulation. Brain. (2000) 123:572–84. 10.1093/brain/123.3.572 10686179

[22] Calabrò RS Naro A Russo M Bramanti P Carioti L Balletta T. Shaping neuroplasticity by using powered exoskeletons in patients with stroke: a randomized clinical trial. J Neuroeng Rehabil. (2018) 15:35. 10.1186/s12984-018-0377-8 29695280 PMC5918557

[23] Demeyere N Riddoch MJ Slavkova ED Bickerton WL Humphreys GW. The Oxford Cognitive Screen (OCS): validation of a stroke-specific short cognitive screening tool. Psychol Assess. (2015) 27:883–94. 10.1037/pas0000082 25730165

[24] Mancuso M Varalta V Sardella L Capitani D Zoccolotti P Antonucci G. Italian normative data for a stroke specific cognitive screening tool: the Oxford Cognitive Screen (OCS). Neurol Sci. (2016) 37:1713–21. 10.1007/s10072-016-2650-6 27395388

[25] Smith A Symbol Digit Modalities Test. Western Psychological Services Los Angeles (1973).

[26] Nocentini U Giordano A Di Vincenzo S Panella M Pasqualetti P. The symbol digit modalities test - Oral version: Italian normative data. Funct Neurol. (2006) 21:93–6. 16796824

[27] Hebb DO. Distinctive features of learning in the higher animal. In: Delafresnaye JF, editor. Brain Mechanisms and Learning. London: Oxford University Press (1961). p.37–46.

[28] Monaco M Costa A Caltagirone C Carlesimo GA. Forward and backward span for verbal and visuo-spatial data: standardization and normative data from an Italian adult population. Neurol Sci. (2013) 34:749–54. 10.1007/s10072-012-1130-x 22689311

[29] Rey A. L'examen psychologique dans les cas d'encéphalopathie traumatique. Arch Psychol. (1941) 28:215–85.16987634

[30] Osterrieth PA Le test de copie d'une figure complexe; contribution à l'étude de la perception et de la mémoire. Arch Psychol. (1944) 30:206–356.

[31] Shin MS Park SY Park SR Seol SH Kwon JS. Clinical and empirical applications of the Rey-Osterrieth complex figure test. Nat Protoc. (2006) 1:892–99. 10.1038/nprot.2006.115 17406322

[32] Meyers JE Meyers KR. Rey Complex Figure Test and Recognition Trial Professional Manual. Psychological Assessment Resources (1995).

[33] Shallice T. Specific impairments of planning. Philos Trans R Soc London B. (1982) 298:199–209. 10.1098/rstb.1982.0082 6125971

[34] Unterrainer JM Rahm B Kaller CP Leonhart R Quiske K Hoppe-Seyler K Meier C Müller C Halsband U. Planning abilities and the Tower of London: is this task measuring a discrete cognitive function? J Clin Exp Neuropsychol. (2004) 26:846–56. 10.1080/13803390490509574 15370380

[35] Berg WK Byrd DL The Tower of London spatial problem-solving task: enhancing clinical and research implementation. J Clin Exp Neuropsychol. (2002) 24:586–604. 10.1076/jcen.24.5.586.1006 12187443

[36] Boccia M Marin D D'Antuono G Ciurli P Incoccia C Antonucci G. The tower of London (ToL) in Italy: standardization of the ToL test in an Italian population. Neurol Sci. (2017) 38:1263–70. 10.1007/s10072-017-2957-y 28432516

[37] Caffarra P Vezzadini G Dieci F Zonato F Venneri A A short version of the Stroop test: normative data in an Italian population sample. Nuova Riv di Neurol. (2002) 12:111–5.

[38] Fugl-Meyer AR Jääskö L Leyman I Olsson S Steglind S. The post-stroke hemiplegic patient. 1. a method for evaluation of physical performance. Scand J Rehabil Med. (1975) 7:13–31. 1135616

[39] W Bohannon R Motricity index scores are valid indicators of paretic upper extremity strength following stroke. J Phys Ther Sci. (1999) 11:59–61. 10.1589/jpts.11.59

[40] Shah S Vanclay F Cooper B. Improving the sensitivity of the Barthel Index for stroke rehabilitation. J Clin Epidemiol. (1989) 42:703–9. 10.1016/0895-4356(89)90065-6 2760661

[41] Jakob I Kollreider A Germanotta M Benetti F Cruciani A Padua L Aprile I. Robotic and sensor technology for upper limb rehabilitation. PM&R. (2018) 10:S189–97. 10.1016/j.pmrj.2018.07.011 30269805

[42] Aprile I Cruciani A Germanotta M Gower V Pecchioli C Cattaneo D Upper limb robotics in rehabilitation: an approach to select the devices, based on rehabilitation aims, and their evaluation in a feasibility study. Appl Sci. (2019) 9:3920 10.3390/app9183920

[43] Woytowicz EJ Rietschel JC Goodman RN Conroy SS Sorkin JD Whitall J. Determining levels of upper extremity movement impairment by applying a cluster analysis to the fugl-meyer assessment of the upper extremity in chronic stroke. Arch Phys Med Rehabil. (2017) 98:456–62. 10.1016/j.apmr.2016.06.023 27519928 PMC5299057

[44] Bernhardt J Borschmann KN Kwakkel G Burridge JH Eng JJ Walker MF. Setting the scene for the Second Stroke Recovery and Rehabilitation Roundtable. Int J Stroke. (2019) 14:450–6. 10.1177/1747493019851287 31092153

[45] Loetscher T Potter KJ Wong D das Nair R. Cognitive rehabilitation for attention deficits following stroke. Cochrane Database Syst Rev. (2019) 2019:CD002842. 10.1002/14651858.CD002842.pub3 31706263 PMC6953353

[46] Maggio MG Latella D Maresca G Sciarrone F Manuli A Naro A. Virtual reality and cognitive rehabilitation in people with stroke: an overview. J Neurosci Nurs. (2019) 51:101–5. 10.1097/JNN.0000000000000423 30649091

[47] De Luca R Lo Buono V Leo A Russo M Aragona B Leonardi S. Use of virtual reality in improving poststroke neglect: promising neuropsychological and neurophysiological findings from a case study. Appl Neuropsychol. (2019) 26:96–100. 10.1080/23279095.2017.1363040 28937807

[48] Zinn S Dudley TK Bosworth HB Hoenig HM Duncan PW Horner RD. The effect of poststroke cognitive impairment on rehabilitation process and functional outcome. Arch Phys Med Rehabil. (2004) 85:1084–90. 10.1016/j.apmr.2003.10.022 15241754

[49] Bui KD Johnson MJ. Robot-based measures of upper limb cognitive-motor interference across the HIV-stroke spectrum. In: IEEE International Conference on Rehabilitation Robotics. (2019) p. 530–5. 31374684 10.1109/ICORR.2019.8779418

[50] Hsieh YW Wu CY Lin KC Yao G Wu KY Chang YJ. Dose-response relationship of robot-assisted stroke motor rehabilitation: the impact of initial motor status. Stroke. (2012) 43:2729–34. 10.1161/STROKEAHA.112.658807 22895994

[51] Adomavičiene A Daunoravičiene K Kubilius R VarŽaityte L Raistenskis J. Influence of new technologies on post-stroke rehabilitation: a comparison of Armeo spring to the kinect system. Med. (2019) 55:98. 10.3390/medicina55040098 30970655 PMC6524064

[52] Zengin-Metli D Ozbudak-Demir S Eraktas I Binay-Safer V Ekiz T. Effects of robot assistive upper extremity rehabilitation on motor and cognitive recovery, the quality of life, and activities of daily living in stroke patients. J Back Musculoskelet Rehabil. (2018) 31:1059–64. 10.3233/BMR-171015 29966188

[53] Hsiao PC Yu WH Lee SC Chen MH Hsieh CL. Responsiveness and predictive validity of the tablet-based symbol digit modalities test in patients with stroke. Eur J Phys Rehabil Med. (2019) 55:29–34. 10.23736/S1973-9087.18.05210-3 29904048

